# Metabolic engineering to enhance the accumulation of bioactive flavonoids licochalcone A and echinatin in *Glycyrrhiza inflata* (Licorice) hairy roots

**DOI:** 10.3389/fpls.2022.932594

**Published:** 2022-08-18

**Authors:** Zhigeng Wu, Sanjay Kumar Singh, Ruiqing Lyu, Sitakanta Pattanaik, Ying Wang, Yongqing Li, Ling Yuan, Yongliang Liu

**Affiliations:** ^1^Key Laboratory of South China Agricultural Plant Molecular Analysis and Genetic Improvement and Guangdong Provincial Key Laboratory of Applied Botany, South China Botanical Garden, Chinese Academy of Sciences, Guangzhou, China; ^2^University of Chinese Academy of Sciences, Beijing, China; ^3^Department of Plant and Soil Sciences and Kentucky Tobacco Research and Development Center, University of Kentucky, Lexington, KY, United States

**Keywords:** *Glycyrrhiza inflata*, flavonoids, echinatin, licochalcone A, AtMYB12 gene, metabolic engineering

## Abstract

Echinatin and licochalcone A (LCA) are valuable chalcones preferentially accumulated in roots and rhizomes of licorice (*Glycyrrhiza inflata*). The licorice chalcones (licochalcones) are valued for their anti-inflammatory, antimicrobial, and antioxidant properties and have been widely used in cosmetic, pharmaceutical, and food industries. However, echinatin and LCA are accumulated in low quantities, and the biosynthesis and regulation of licochalcones have not been fully elucidated. In this study, we explored the potential of a R2R3-MYB transcription factor (TF) *AtMYB12*, a known regulator of flavonoid biosynthesis in *Arabidopsis*, for metabolic engineering of the bioactive flavonoids in *G. inflata* hairy roots. Overexpression of *AtMYB12* in the hairy roots greatly enhanced the production of total flavonoids (threefold), echinatin (twofold), and LCA (fivefold). RNA-seq analysis of *AtMYB12*-overexpressing hairy roots revealed that expression of phenylpropanoid/flavonoid pathway genes, such as *phenylalanine ammonia-lyase* (*PAL*), *chalcone synthase* (*CHS*), and *flavanone 3’-hydroxylase* (*F3’H*), is significantly induced compared to the control. Transient promoter activity assay indicated that AtMYB12 activates the *GiCHS1* promoter in plant cells, and mutation to the MYB-binding motif in the *GiCHS1* promoter abolished activation. In addition, transcriptomic analysis revealed that *AtMYB12* overexpression reprograms carbohydrate metabolism likely to increase carbon flux into flavonoid biosynthesis. Further, AtMYB12 activated the biotic defense pathways possibly by activating the salicylic acid and jasmonic acid signaling, as well as by upregulating WRKY TFs. The transcriptome of *AtMYB12*-overexpressing hairy roots serves as a valuable source in the identification of potential candidate genes involved in LCA biosynthesis. Taken together, our findings suggest that *AtMYB12* is an effective gene for metabolic engineering of valuable bioactive flavonoids in plants.

## Introduction

*Glycyrrhiza* species of the family Fabaceae, including *Glycyrrhiza glabra* L., *Glycyrrhiza uralensis* Fisch., and *Glycyrrhiza inflata* Bat., are valued greatly for their roots and rhizomes (licorice), which are widely used in cosmetics and herbal medicines (Zhang and Ye, 2009; Jiang et al., 2020). The bioactivity of licorice is mainly attributed to two groups of specialized metabolites:, namely, triterpene saponins and flavonoids (Wang D. et al., 2020; Wang Z.-F. et al., 2020). Glycyrrhizin is the most abundant saponin in licorice and has long been recognized as a potent sweetening agent (Pandey and Ayangla, 2018). Glycyrrhizin also has been explored for anti-coronavirus properties in the current COVID-19 pandemic (Luo et al., 2020; Chrzanowski et al., 2021). The other major group of bioactive components present in licorice are flavonoids (Zhu et al., 2016; Cheng et al., 2021). The licorice flavonoids are known to possess anti-inflammatory, antioxidant, and antimicrobial properties (Wang Z.-F. et al., 2020; Husain et al., 2021). Among the different flavonoids, echinatin and licochalcone A (LCA) are predominantly present in *G. inflata* (Lin et al., 2017; Rizzato et al., 2017; Song et al., 2017). A high LCA cosmetic formulation reduces UV-induced erythema formation in human healthy volunteers possibly by modulation of dendritic cell activity (Kolbe et al., 2006). Because of its anti-inflammatory and antimicrobial properties, LCA has been used for the treatment of facial skin diseases such as acne and rosacea (Schoelermann et al., 2016; Yang et al., 2018). Therefore, there is a great demand of LCA in cosmetic industries (Nguyen et al., 2020; Cerulli et al., 2022). However, LCA is naturally accumulated at low levels in wild *G. inflata*, even less in cultivated *G. inflata* plants. Metabolic engineering is thus viewed as a rational alternative to increase LCA production.

**FIGURE 1:**
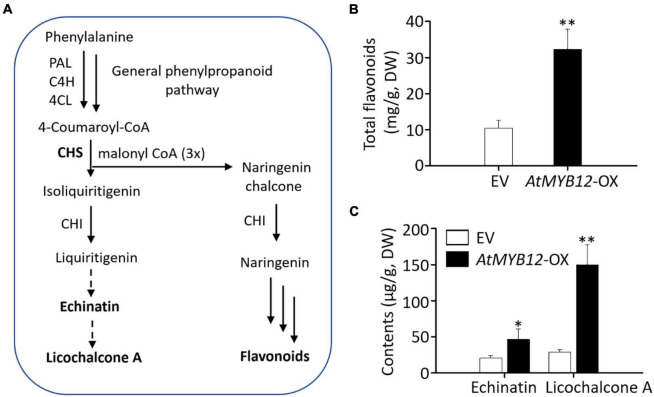
Proposed biosynthetic pathway of licorice chalcones and measurement of metabolites in empty vector (EV) control and *AtMYB12*-OX hairy roots of *G. inflata*. **(A)** A simplified, schematic diagram of the proposed licorice chalcone and general flavonoid biosynthetic pathway. PAL, phe ammonia lyase; C4H, cinnamate 4-hydroxylase; 4CL, 4-coumarate; CoA ligase; CHS, chalcone synthase; CHI, chalcone isomerase. Solid arrows indicate known enzymes; dotted arrows indicate pathway enzymes are not known. **(B,C)** Echinatin and licochalcone A contents on dry weight basis. Data are presented as the mean ± SD (*n* = 3). Asterisks indicate statistically significant differences compared with EV lines (*p < 0.05, ^**^p < 0.01, Student’s *t*-test). Total flavonoids based on dry weight (DW).

Chalcones are a subgroup of polyphenol compounds that are synthesized through the phenylpropanoid pathway (Figure 1A). The precursor phenylalanine (Phe) is derived from the primary metabolic pathways, including glycolysis, the shikimate pathway, and Phe biosynthetic pathway (Tzin and Galili, 2010). Chalcone synthase (CHS) is the first rate-limiting enzyme specific for flavonoid pathway (Saito et al., 2013). Regulation of the flavonoid biosynthesis has been extensively studied in numerous plant species including *Arabidopsis thaliana* (Saito et al., 2013). In *Arabidopsis*, three closely related MYB transcription factors (TFs), MYB11, MYB12, and MYB111, from subgroup 7 of the R2R3-MYB family, redundantly regulate the biosynthesis of flavonoids, especially flavonols (Mehrtens et al., 2005; Stracke et al., 2007). These MYBs bind to the promoters of key flavonoid biosynthetic pathway genes, such as *CHS*, to activate expression (Mehrtens et al., 2005; Stracke et al., 2007). The three MYBs exhibit distinct expression patterns, and AtMYB12 mainly controls flavonoid biosynthesis in *Arabidopsis* roots (Stracke et al., 2007). The regulatory role of AtMYB12 on flavonoid pathway has been further investigated through heterologous expression in tobacco leaves and tomato fruits (Luo et al., 2008; Pandey et al., 2015; Zhang et al., 2015). AtMYB12 induces the accumulation of flavonoids in tomato fruits by reprogramming the primary metabolism and directing the carbon flux toward flavonoid pathway (Zhang et al., 2015). Chlorogenic acid (CGA) is a subclass of polyphenols present in *Solanaceous* species (tomato and tobacco) and coffee, but not in *Arabidopsis* (Luo et al., 2008; Naveed et al., 2018). In addition to flavonoids, ectopic expression of *AtMYB12* in tobacco significantly increases CGA biosynthesis (Luo et al., 2008; Zhang et al., 2015). AtMYB12 also activates CGA biosynthetic genes in tomato fruits (Luo et al., 2008; Zhang et al., 2015). AtMYB12 overexpression in kale increases total flavonoid and phenolics in leaves (Lännenpää, 2014). These findings suggest that AtMYB12 is a potential candidate for metabolic engineering to induce flavonoids and flavonoid-derived metabolites in heterologous plant species.

As the biosynthetic pathway and gene regulation of licorice chalcones are not well elucidated, we aimed to explore the potential of AtMYB12 for metabolic engineering of licorice chalcones in *G. inflata*. We hypothesized that ectopic expression of *AtMYB12* in *G. inflata* will lead to higher accumulation of licorice chalcones and identification of potential chalcone pathway genes. As protocol for the generation of stable transgenic lines is not established in *G. inflata*, we thus generated hairy roots overexpressing *AtMYB12*. Molecular and biochemical analyses of *AtMYB12*-overexpressing hairy roots showed higher expression of phenylpropanoid/flavonoid pathway genes, including *GiCHS*, and increased accumulation of total flavonoids and licorice-specific flavonoids, such as echinatin and LCA, confirming the regulatory roles of AtMYB12 on early flavonoid pathway genes in a heterogeneous plant species. In addition, RNA-seq data showed that the carbon flux was reprogrammed toward the flavonoid pathway. Our findings suggest that AtMYB12 is an effective regulator for engineering the production of licorice chalcones in *G. inflata*.

## Materials and methods

### Plant materials

*Arabidopsis thaliana* Col-0 accession was used for RNA isolation and *AtMYB12* cloning. *G. inflata* seeds were provided by Gansu Jin You Kang Pharmaceutical Technology Co., Ltd., Lanzhou, China. *G. inflata* seeds were soaked in H_2_SO_4_ for 30 min, washed with water five times, then treated with 1% NaClO for 10 min, and washed with sterilized distilled water five times. Surface-sterilized seeds were germinated on Murashige and Skoog (MS) medium and kept in dark for 2 days before being transferred to light condition. Then, 8-day-old *G. inflata* seedlings were used for DNA, RNA isolation, transient gene expression, and generation of transgenic hairy roots.

### Generation of transgenic *AtMYB12* hairy roots

*AtMYB12* was amplified from *Arabidopsis* cDNA and cloned into the pCAMBIA2301 vector containing *CaMV*35S promoter and *rbcS* terminator to generate pCAMBIA2301-AtMYB12. Primers used for cloning of *AtMYB12* and other genes in this study are all listed in Supplementary Table 1. The empty vector (EV; pCAMBIA2301) and pCAMBIA2301-AtMYB12 plasmids were separately transformed into *Agrobacterium rhizogenes* R1000 by freeze–thaw method. The hypocotyl segments from 8-day-old *G. inflata* seedlings were submerged in the *A. rhizogenes* R1000 suspension for 30 min, blot-dried on sterile filter paper, and then placed on MS medium at 22^°^C in darkness. After co-cultivation for 2 days, the hypocotyl segments were transferred to MS medium supplemented with 400 mg/l cefotaxime. After 2–3 weeks of culture, hairy roots developed from hypocotyls and the rapidly growing hairy roots were excised and cultivated individually on solid MS medium supplemented with 400 mg/l cefotaxime and 100 mg/l kanamycin for 2 weeks at 25^°^C in dark. Rapidly growing root lines that showed kanamycin resistance were selected for further analysis. These hairy root lines were cultured in 125 ml flasks each containing 10 ml MS liquid medium on an orbital shaker at 100 rpm at 25^°^C. The hairy roots clones were routinely subcultured every 2 weeks and harvested after 2 months for RNA isolation and metabolite extraction.

### cDNA synthesis and determination of transgenic status of the hairy roots

Total RNA was isolated from EV control and *AtMYB12*-overexpressing seedlings and hairy roots using the RNeasy Plant Mini Kit following the instructions of the manufacturer (QIAGEN, United States). Approximately 2 μg of total RNA was used for DNase I digestion. Synthesis of first-strand cDNA was performed using Superscript III reverse transcriptase (Invitrogen) in a total volume of 20 μl. To verify the transgenic status of *AtMYB12*-OX and EV control hairy root lines, gene-specific primers were used to PCR-amplify the *rol B*, *rol C*, *vir C*, and kanamycin-resistant (*nptII*) genes. PCR products were analyzed on a 1% ethidium bromide-stained agarose gel.

### Determination of the contents of flavonoids in hairy roots

Total flavonoid contents were determined by sodium nitrite–aluminum nitrate colorimetric method using rutin as standard (Hao et al., 2018). The standards rutin, echinatin, and LCA were purchased from Biosynth Carbosynth, United States. The contents of echinatin and LCA were determined by LC-MS/MS.

### Library construction and RNA sequencing

Three independent lines of both EV and *AtMYB12*-OX hairy roots were used for RNA-seq. Total RNA was isolated from hairy roots using the RNeasy Plant Mini Kit (QIAGEN, United States) following the instructions of the manufacturer. The RNA samples with RNA integrity number (RIN) 8 or above were used for library preparation and sequencing. The TruSeq RNA Sample Prep Kit (Illumina, United States) was used for making libraries according to the protocol of the manufacturer. Individually indexed libraries were combined at equal proportions and loaded onto a single lane of a flow cell. A 50-cycle single-end sequencing run was performed on the Illumina HiSeq2500 at the Duke Center for Genomic and Computational Biology.

### Data processing, identification of differentially expressed genes, and gene ontology enrichment analysis

Raw Illumina sequence reads were processed as described previously (Singh et al., 2015). Read mapping was performed by Bowtie2 (Langmead and Salzberg, 2012) using an in-house-generated *G. inflata* transcriptome (unpublished data). Differential gene expression analysis was carried out using the DESeq2 Bioconductor package in R (Love et al., 2014). The differentially expressed genes (DEGs) were identified following two criteria: (i) fold change ≥ 2 and (ii) false discovery rate *p*-value correction of ≤ 0.05. Heatmaps were constructed using the Complex Heatmap (Gu et al., 2016) function in R through the Bioconductor package (R Core Team, 2022). Functional annotation of DEGs was performed with eggNOG 4.5 (Huerta-Cepas et al., 2016) database. Gene Ontology (GO) analysis of the enriched functional categories was performed using BiNGO (version 2.44) (Maere et al., 2005).

### Reverse transcription quantitative PCR

Reverse transcription quantitative PCR (RT-qPCR) was used to measure transcripts levels of *GiCHS* genes. The *GiActin* gene was used as an internal control. Relative gene expression was measured as previously described (Liu et al., 2019). All qRT-PCRs were performed in triplicate and repeated twice. Primers used in qRT-PCR are listed in Supplementary Table 1.

### Transient overexpression of *AtMYB12* in *Glycyrrhiza inflata* seedlings

The EV and pCAMBIA2301-AtMYB12 were transformed into *Agrobacterium tumefaciens* GV3101 by freeze–thaw method and was plated on Luria–Bertani (LB) medium containing 100 μg ml^–1^ kanamycin, 50 μg ml^–1^ gentamicin, and 30 μg ml^–1^ rifampicin. A single colony was transferred to 1 ml liquid LB medium containing the same antibiotics and incubated at 250 rpm and 28°C overnight. The overnight culture was diluted in 25 ml liquid LB medium and grown for 16 h at 250 rpm and 28°C. The cells were then centrifuged, and the pellet was resuspended in infiltration buffer (10 mM MgCl_2_, 10 mM MES, 100 μM acetosyringone) to an OD_600_ of 1.0, and incubated at 28°C for at least 3 h. Then, 8-day-old *G. inflata* seedlings were immersed in the infiltration solution under vacuum pressure for 1 h. After vacuum infiltration, seedlings were washed five times with sterile distilled water and laid on sterile wet filter papers in Petri dishes. After 5 days of incubation at room temperature, the transfected seedlings were collected for RNA isolation.

### Cloning of the *GiCHS1* promoter

Genomic DNA was extracted from *G. inflata* seedlings for promoter cloning. A forward primer (CHS1-pro-F) was designed based on the genomic sequence upstream of the coding region of *G. uralensis* homolog of *GiCHS1*. A reverse primer (GiCHS1-cds-R) was designed within the coding sequence of *GiCHS1*. PCR product of GuCHS1-pro-F and GiCHS1-cds-R was sequenced. *CHS1* promoter sequences of *G. inflata* and *G. uralensis* were aligned using ClustalW software (Thompson et al., 2003). Based on the *GiCHS1* promoter sequence, another pair of primers (GiCHS1-pro-F2 and GiCHS1-pro-R2) was designed for vector construction.

### Promoter activity assay in tobacco protoplasts

Tobacco cell line described before (Pattanaik et al., 2010) was used for protoplast isolation and promoter activity assay. The effector plasmid was constructed by cloning *AtMYB12* into a modified pBS vector under the control of the *CaMV*35S promoter and *rbcS* terminator. The reporter plasmid was generated by cloning *GiCHS1* promoter in a modified pUC vector containing the firefly *luciferase* (*LUC*) reporter and *rbcS* terminator. The MYB binding motif in *GiCHS1* promoter was mutated by site-directed mutagenesis to generate mutant promoter. The *GUS* reporter driven by *CaMV*35S promoter and *rbcS* terminator was used as an internal control in the protoplast assay. The reporter, effector, and internal control plasmids were electroporated into tobacco protoplasts in different combinations; luciferase and GUS activities in transfected protoplasts were measured as described previously (Pattanaik et al., 2010).

## Results

### Ectopic expression of *AtMYB12*-induced total flavonoids and licorice-specific chalcones in *Glycyrrhiza inflata* hairy roots

We generated transgenic hairy roots overexpressing *AtMYB12* (*AtMYB12*-OX) aiming to increase flavonoid production. EV hairy root lines (EV-1, EV-2, and EV-3) served as control. Three independent *AtMYB12*-OX hairy root lines (*AtMYB12*-OX-1, *AtMYB12*-OX-2, and *AtMYB12*-OX-3) were selected for further analysis. The transgenic status of the independent EV and *AtMYB12*-OX hairy root lines was verified by PCR (Supplementary Figure 1). Total flavonoid contents of the three *AtMYB12*-OX lines were significantly higher (threefold) than those of the EV lines (Figures 1A,B). While echinatin and LCA in EV hairy root lines were approximately 18–24 and 25–31 ng mg^–1^, respectively (Figures 1A,C); *AtMYB12*-OX lines showed a significant increase in the accumulation of echinatin (30–59 ng mg^–1^; ∼2.2-fold increase) and LCA (119–174 ng mg^–1^; ∼5.2-fold increase) (Figure 1C). The metabolic outcomes of *AtMYB12* overexpression suggest that *AtMYB12* is an effective gene for metabolic engineering of the licorice flavonoid pathway.

### *AtMYB12*-induced expression of phenylpropanoid/flavonoid pathway genes in *Glycyrrhiza inflata* hairy roots

**FIGURE 2:**
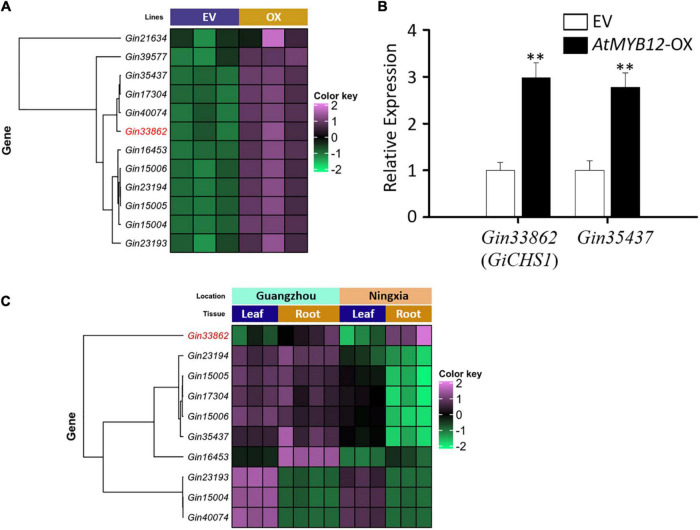
Expression profiles of the *G. inflata CHS* genes induced by AtMYB12. **(A)** Heatmap shows the expression patterns of 12 *GiCHS* genes in EV and *AtMYB12*-OX (OX) hairy root lines. *Gin33862* (*GiCHS1*) is highlighted in red. **(B)** Relative expression of two selected *GiCHS* genes in EV and *AtMYB12*-OX hairy root lines measured by qRT-PCR. Values were normalized to the expression level of an internal control, the *actin* gene (*GiActin*). Data presented are the means of three biological replicates ± SD (*n* = 3). Asterisks indicate statistically significant differences compared with EV lines (^**^p < 0.01, Student’s *t*-test). **(C)** Heatmap shows the expression patterns of 10 *GiCHS* genes in two tissues (root and leaf) collected from two locations (Guangzhou and Ningxia) in China.

The metabolic outcomes of *AtMYB12*-OX hairy roots prompted us to generate and analyze the transcriptome data of EV and *AtMYB12*-OX lines. Sequencing of RNA libraries of EV and *AtMYB12*-OX lines generated a total of 1,110 million (M) clean reads. Each biological replicate was represented by an average of more than 170 M reads. On average, more than 70% of the total reads from EV and overexpression line libraries were successfully mapped to the *G. inflata* transcriptome (Supplementary Figure 2). Compared to the EV lines, 3,236 genes were differentially expressed in *AtMYB12*-OX lines, in which 1,722 genes were upregulated and 1,514 genes were downregulated (Supplementary Table 2). We particularly examined genes in the phenylpropanoid/flavonoid pathway. CHS is a key rate-limiting enzyme in flavonoid biosynthetic pathway (Zhang et al., 2017). Noticeably, 13 *GiCHSs* were identified among the DEGs, and 12 of them were upregulated in *AtMYB12*-OX hairy roots (Figure 2A and Table 1). In addition, we identified three *G. inflata phenylalanine ammonia-lyase* (*PAL*) and 9 *flavanone 3’-hydroxylase* (*F3’H*) genes among the DEGs, and all of them were induced in *AtMYB12*-OX hairy roots (Table 1). To verify the expression of selected DEGs in RNA-seq, we conducted RT-qPCR to measure the expression of two *CHS* genes, *Gin33862* (hereafter designed as *GiCHS1*) and *Gin35437*, using independently isolated RNAs from the *AtMYB12*-OX hairy roots. The results confirmed the induction of both *CHS* genes in *AtMYB12*-OX hairy root lines (Figure 2B). These results suggest that the upregulation of *GiPALs*, *GiCHSs*, and *GiF3’Hs* likely leads to the enrichment of flavonoids in *G. inflata* hairy roots.

**TABLE 1:** Phenylpropanoid/flavonoid pathway genes identified in DEGs.

*Glycyrrhiza inflata* gene ID	Log2 fold change	Arabidopsis homolog	Description
Gin06540	1.618	AT2G37040	Phenylalanine
Gin12084	2.728	AT2G37040	ammonia-lyase (PAL)
Gin12083	2.426	AT3G10340	

Gin31596	–2.659	AT5G13930	
Gin15004	1.081	AT5G13930	
Gin15005	1.223	AT5G13930	
Gin15006	1.121	AT5G13930	
Gin16453	2.211	AT5G13930	
Gin17304	1.242	AT5G13930	
Gin21634	1.167	AT5G13930	Chalcone synthase
Gin23193	1.166	AT5G13930	(CHS)
Gin23194	1.258	AT5G13930	
Gin33862	1.156	AT5G13930	
Gin35437	1.139	AT5G13930	
Gin39577	1.862	AT5G13930	
Gin40074	1.627	AT5G13930	

Gin02931	1.589	AT5G07990	
Gin09638	3.125	AT5G07990	
Gin09639	4.233	AT5G07990	
Gin09641	3.840	AT5G07990	Flavanone
Gin13176	4.344	AT5G07990	3′-hydroxylase (F3′H)
Gin13177	4.586	AT5G07990	
Gin13178	4.038	AT5G07990	
Gin13179	4.270	AT5G07990	
Gin13180	3.766	AT5G07990	

### *GiCHS1* is highly expressed in roots

LCA and echinatin are preferentially accumulated in *G. inflata* roots and rhizomes. We therefore analyzed the transcriptomes (SRA accession: PRJNA574093) of *G. inflata* leaves and roots, collected from two geographical locations in China (Guangzhou and Ningxia), to determine the tissue-specific expression of *CHS*. Among the 12 *GiCHSs* upregulated in *AtMYB12-OX* roots, expression of two *GiCHSs* was not detected in leaf and root transcriptomes. Among the other 10 *GiCHSs*, *Gin33862* (*GiCHS1*) is preferentially expressed in *G. inflata* roots from both locations (Figure 2C). Two other *GiCHSs*, *Gin35437* and *Gin16453*, showed increased expression only in the roots collected from Guangzhou (Figure 2C).

### Transient overexpression of *AtMYB12* in *Glycyrrhiza inflata* seedlings induced *GiCHSs* expression

**FIGURE 3:**
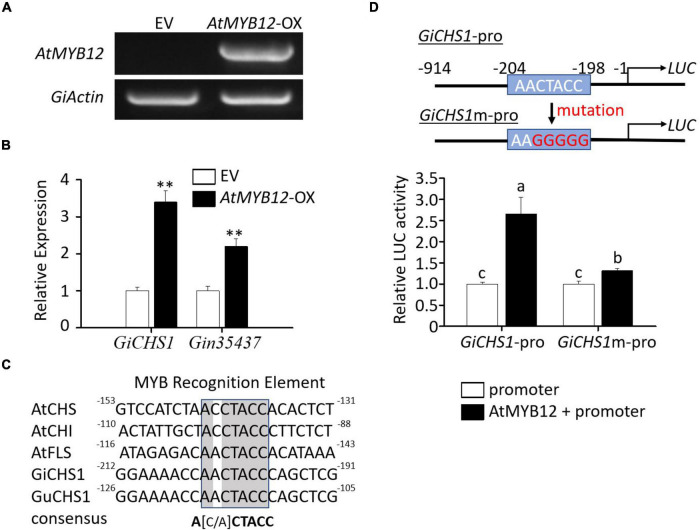
Molecular analysis of *G. inflata* seedlings transiently overexpressing *AtMYB12* and transactivation assay of the *GiCHS1* promoter. **(A)** RT-PCR analysis showed *AtMYB12* expression in *G. inflata AtMYB12*-OX seedlings but not in EV seedlings. The *G. inflata* actin gene (*GiActin*) served as internal control. **(B)** Relative expression of the two *GiCHS* genes in *AtMYB12*-OX seedlings was measured using qRT-PCR. *GiActin* was used as an internal control for normalization. Data are presented as the mean of three biological replicates ± SD. Asterisks indicate statistically significant differences compared with EV lines (^**^p < 0.01, Student’s *t*-test). **(C)** Similar to the promoters of *Arabidopsis CHS*, *CHI*, and *FLS*, *GiCHS1* and *GuCHS1* promoters also contain the MYB recognition elements (MRE). Numbers by the ends of the DNA sequences, such as -130 at the right end of *AtCHS* promoter sequence, represent positions relative to translation start site. **(D)** The diagram in the right shows the MRE in the *GiCHS1* promoter (*GiCHS1*-pro) and the mutated MRE sequence in the mutant promoter (*GiCHS1m*-pro). *LUC*, the reporter *luciferase* gene. Left panel shows transactivation of *GiCHS1*-pro and *GiCHS1m*-pro after infiltration of the promoter vector alone or in combination with the AtMYB12-expression vector into tobacco cells. Data presented as the mean of biological replicates ± SD (*n* = 3). Asterisks indicate statistically significant differences compared with EV lines (^**^p < 0.01, Student’s *t*-test).

To further verify the effect of AtMYB12 on LCA biosynthesis in *G. inflata*, we developed an *Agrobacteria-*mediated transient gene expression assay in *G. inflata* seedlings. Similar to that in the hairy roots, expression of *GiCHS1* and *Gin35437* was significantly induced by ectopic expression of *AtMYB12* in *G. inflata* seedlings (Figures 3A,B). These results indicate that the heterologous AtMYB12 positively regulates flavonoid biosynthesis in *G. inflata* plants.

### *AtMYB12* directly activates the *GiCHS1* promoter activity

We next asked whether AtMYB12 directly activates the flavonoid pathway gene promoters in *G. inflata*. As *GiCHS1* expression is upregulated by AtMYB12 and highly expressed in roots, we cloned the *GiCHS1* promoter for activity assay. Due to the lack of genomic sequences for *G. inflata*, the *GiCHS1* promoter was cloned based on the *G. uralensis* genome sequences (Mochida et al., 2017) as *G. inflata* and *G. uralensis* are two closely related species. The amino acid sequence identity between *GiCHS1* and its *G. uralensis* homolog is 99%. The promoter of *GiCHS1* also shares high sequence identity (96%) with that of *G. uralensis CHS1* (Supplementary Figure 3). As shown in Figure 3D, transcriptional activity of the *GiCHS1* promoter (*GiCHS1*-pro) was significantly induced by AtMYB12, suggesting that AtMYB12 directly activates the *GiCHS1* promoter in plant cells. To further confirm the activation of the *GiCHS1* promoter by AtMYB12, we surveyed the promoter sequence for MYB recognition element (MRE) (A[A/C]CTACC) and identified a putative MRE (AACTACC) at -204 to -198 relative to ATG. This MRE is conserved among the *Arabidopsis CHS*, *CHI*, and *FLS* promoters and also present in the *CHS* promoters from other plants (Figure 3C). It is predicted to be targeted by R2R3 MYBs, including MYB11, MYB111, and MYB12 (Stracke et al., 2007). We speculated that this MRE (AACTACC) in the *GiCHS1* promoter is targeted by AtMYB12. We mutated this motif (to AAGGGGG) to generate the mutant *GiCHS1* promoter (*GiCHS1m*-pro) (Figure 3D). Results of promoter activity assay showed that AtMYB12 is unable to activate *GiCHS1m*-pro (Figure 3D), suggesting that AtMYB12 directly binds to the MYB binding site in the *GiCHS1* promoter.

### RNA-seq revealed reprogramming of carbohydrate metabolism in *AtMYB12*-OX lines

**FIGURE 4:**
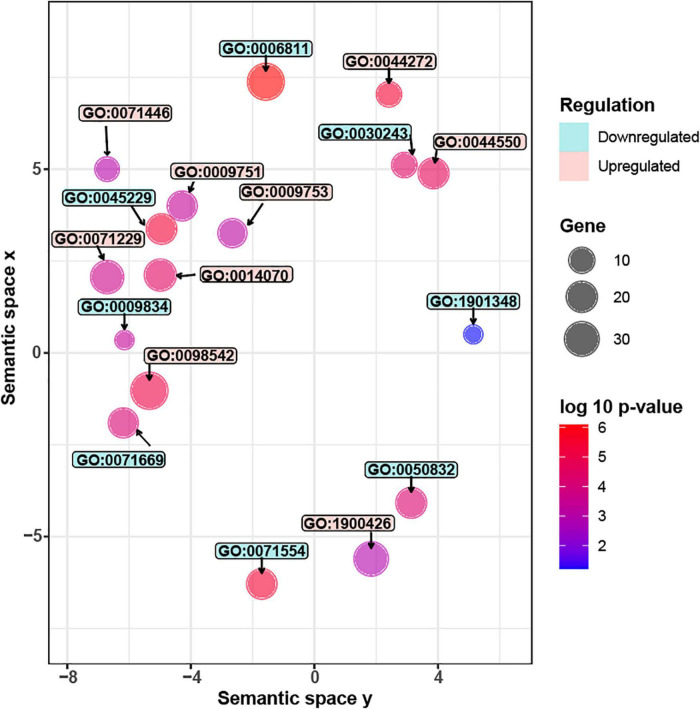
Significantly enriched GO terms in *G. inflata AtMYB12*-OX hairy roots. Gene Ontology (GO) analyses of differentially expressed genes (DEGs). Upregulated GO terms are colored in *red* while downregulated terms are in *blue*. Each circle represents one GO term. The circle size represents the number of genes in each GO category while the color represents the significance level. Description of the upregulated GO terms (from top to bottom): GO:0042742, defense response to bacterium; GO:0071446, cellular response to salicylic acid stimulus; GO:0044550, secondary metabolite biosynthetic process; GO:0009751, response to salicylic acid; GO:0009753, response to jasmonic acid; GO:0071229, cellular response to acid chemical; GO:0014070, response to organic cyclic compound; GO:0098542, defense response to other organism; GO:1900426, positive regulation of defense response to bacterium. Description of the downregulated GO terms (from top to bottom): GO:0006811, ion transport; GO:0030243, cellulose metabolic process; GO:0045229, external encapsulating structure organization; GO:0009834, plant-type secondary cell wall biogenesis; GO:1901348, positive regulation of secondary cell wall biogenesis; GO:0071669, plant-type cell wall organization or biogenesis; GO:0050832, defense response to fungus; GO:0071554, cell wall organization or biogenesis.

Carbon resources of phenylpropanoids are derived from monosaccharides, such as glucose. The monosaccharides are directed to phenylpropanoid pathway through several primary pathways, including pentose phosphate pathway, glycolysis, and the shikimate pathway (Zhang et al., 2015). We observed that three genes related to glycolysis and shikimate pathways were induced by AtMYB12 in *G. inflata* hairy roots (Supplementary Table 3). Glucose also serves as a precursor of the polysaccharide cellulose, the major component of plant cell wall (Taylor, 2008; Yang et al., 2018). Further, GO enrichment analysis (Figure 4 and Supplementary Table 4) showed that several pathway genes related to cellulose synthesis and cell wall synthesis, including “cell wall biogenesis” and “cellulose metabolic process,” are downregulated in *AtMYB12*-OX lines. Cellulose production during cell wall biosynthesis has been shown to be dependent on cellulose synthase A (CESA). We identified seven *G. inflata CESA* genes in the DEGs that are downregulated in *AtMYB12*-OX lines (Table 2). These results indicate an increased carbon flux toward the phenylpropanoid pathway at the cost of cellulose synthesis in *AtMYB12*-OX hairy roots.

**TABLE 2:** *Cellulose synthase A* (*CESA*) genes identified in DEGs.

*Glycyrrhiza inflata* gene ID	Log2 fold change	Arabidopsis homolog	Description
Gin23691	–1.032	AT4G39350	Cellulose synthase A2 (CESA2)
Gin14785	–1.563	AT5G44030	Cellulose synthase A4 (CESA4)
Gin34862	–1.408	AT5G44030	Cellulose synthase A4 (CESA4)
Gin05128	–1.259	AT5G17420	Cellulose synthase A7 (CESA7)
Gin32817	–1.666	AT5G17420	cellulose synthase A7 (CESA7)
Gin06050	–3.691	AT4G18780	Cellulose synthase A8 (CESA8)
Gin19934	–1.230	AT4G18780	Cellulose synthase A8 (CESA8)

### Pathogen defense response genes were activated in *AtMYB12*-OX lines

**FIGURE 5:**
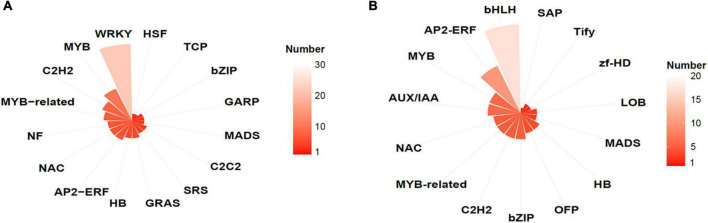
Transcription factor (TF) families identified in DEGs in EV and AtMYB12 transcriptomes. **(A)** Upregulated TFs represented by gene numbers (the lighter the color, the higher the number); e.g., the most upregulated TFs belong to WRKY family. **(B)** Downregulated TFs represented by gene numbers (the lighter the color, the higher the number); e.g., the bHLH family is most affected.

GO enrichment analysis showed that, in *AtMYB12*-OX lines, most of the upregulated pathways are related to pathogen defense responses, including “defense response to bacterium,” “defense response to oomycetes,” “response to fungus,” and “innate immune response” (Figure 4). Salicylic acid (SA) and jasmonic acid (JA) are two important phytohormones that are particularly involved in pathogen defense (Yang et al., 2019; Chen et al., 2020). We observed that the SA and JA signaling pathways, including “response to salicylic acid,” “cellular response to salicylic acid stimulus,” and “response to jasmonic acid,” were activated (Figure 4 and Supplementary Table 4). In addition, a number of TF families were identified among the DEGs. In particular, members of the *WRKY* TFs are enriched in the DEGs (Figure 5). A growing body of research suggests that WRKY TFs are involved in pathogen resistance (Wani et al., 2021). Therefore, it is possible that AtMYB12-mediated defense responses are activated through SA and JA signaling, as well as the activation of WRKY TFs.

## Discussion

TFs are ideal candidates for metabolic engineering because of their broad regulatory roles in metabolic pathways (Broun, 2004; Lu et al., 2016). Increasing evidence suggests that many TF functions and regulatory mechanisms are conserved across the species (Feller et al., 2011; Schluttenhofer and Yuan, 2015). For instance, ectopic expression of the maize bHLH TF *Lc* induces anthocyanin accumulation in tobacco and *Arabidopsis* (Lloyd et al., 1992). Similarly, expression of snapdragon R2R3 MYB *Rosea1* and bHLH TF *Delila*, both driven by a fruit-specific promoter, induces anthocyanin accumulation in tomato fruit (Butelli et al., 2008). Ectopic expression of the *Arabidopsis* R2R3 MYB *PAP1* induces anthocyanin accumulation in tobacco (Borevitz et al., 2000). These findings support the conserved regulatory roles of TFs in metabolic pathways in plants. Licorice chalcones, including echinatin and LCA, are a characteristic group of flavonoids that are exclusively produced in *G. inflata* roots and rhizomes (Lin et al., 2017; Rizzato et al., 2017; Song et al., 2017). Although echinatin and LCA possess several important bioactive properties (Wang D. et al., 2020; Wang Z.-F. et al., 2020), key enzymes involved in the biosynthesis and molecular mechanism of regulation have not been fully elucidated. AtMYB12, along with AtMYB11 and AtMYB111, regulates flavonoid biosynthesis in *Arabidopsis* (Stracke et al., 2007). Heterologous expression of *AtMYB12* in tomato not only activates flavonoid pathway genes but also induces genes in the upstream primary metabolic pathways (Luo et al., 2008; Zhang et al., 2015). Here, we demonstrated that ectopic expression of *AtMYB12* upregulates the expression of *GiCHSs* and enhances echinatin and LCA accumulation in *G. inflata* roots (Figures 1, 3).

A transformation protocol to generate stable transgenic plants is not available for *G. inflata*. As licorice-specific flavonoids are preferentially accumulated in rhizomes and roots of *G. inflata* (Lin et al., 2017; Rizzato et al., 2017; Song et al., 2017), we used transgenic hairy roots to explore the potential of AtMYB12 to enhance the accumulation of echinatin and LCA. RNA-seq analysis of three independent hairy root lines showed upregulation of flavonoid pathway genes, including *PAL*, *CHS*, and *F3’H* (Table 1). CHS belongs to the plant polyketide synthase superfamily and is a key enzyme in the flavonoid pathway (Dao et al., 2011). CHS catalyzes the condensation of one molecule cinnamic acid or its derivatives and three molecules of malonyl Co-A to produce the narigenin chalcone, which serves as a precursor for diverse sets of flavonoids (Dao et al., 2011). Our RNA-seq data showed that expression of 12 *G. inflata CHSs* is upregulated by AtMYB12 (Figure 2A and Table 1).

*Agrobacterium*-mediated transient transformation of whole seedlings has been used to study the regulation of metabolic pathways in different plant species, such as *Catharanthus roseus* (Liu et al., 2019; Mortensen et al., 2019). We transiently transformed *G. inflata* seedlings with *Agrobacterium* harboring *AtMYB12* and measured the expression of selected *CHSs*. Similar to the hairy roots, expression of two selected Gi*CHSs* was significantly induced in seedlings transformed with *AtMYB12* (Figure 3B). We demonstrated that AtMYB12 activates the *GiCHS1* promoter in plant cells by binding to a MYB-recognition element (MRE), and mutation of the MRE abolished the activation (Figure 3D). In *Arabidopsis* (Mehrtens et al., 2005) and tomato (Zhang et al., 2015), AtMYB12 is shown to bind and activate the *CHS* promoter, further reinforcing the functional conservation of AtMYB12.

In tomato fruits, in addition to flavonoids, ectopic expression of *AtMYB12* upregulates CGA biosynthetic pathway genes and increases CGA accumulation (Luo et al., 2008; Zhang et al., 2015). Given the fact that echinatin and LCA contents were higher in *AtMYB12*-OX hairy roots (Figure 1), we speculated that licorice chalcone biosynthetic genes are regulated by AtMYB12. Although genes encoding enzymes in licorice chalcone pathway are not fully characterized, structural differences between echinatin and LCA suggest two modification steps: O-methylation and prenylation likely occur in the biosynthetic steps from echinatin to LCA. From our RNA-seq data set, we identified four genes that are annotated as O-methyltransferases and two as prenyltransferases, all differentially expressed in *AtMYB12*-OX hairy roots (Supplementary Table 5). These genes are thus putative candidates of future investigation of LCA biosynthesis.

In tomato fruits, other than increased accumulation of flavonoids, ectopic expression of *AtMYB12* decreases the contents of carbon resources, including glucose and fructose (Zhang et al., 2015). Genes in the primary metabolic pathways were mostly induced by AtMYB12 (Zhang et al., 2015). Our results confirmed the induction of the genes in the primary pathways upstream of flavonoids (Supplementary Table 3). We also found that the genes related to cellulose synthesis were downregulated (Figure 4 and Table 2), suggesting that, in both tomato fruits (Zhang et al., 2015) and *G. inflata* hairy roots, AtMYB12 redirects carbon flux from other carbohydrate resources toward the flavonoid pathway. Therefore, we suggest that the functionally conserved nature of AtMYB12 makes it a promising candidate for metabolic engineering in other plant species.

*AtMYB12* overexpression improves pathogen resistance in transgenic tobacco plants (Ding et al., 2021). In transgenic tobacco, *AtMYB12* induces the production of flavonoid compounds, such as rutin, as well as reactive oxygen species, H_2_O_2_, and NO (Pandey et al., 2015; Ding et al., 2021). Similar to tobacco, our results revealed that the genes involved in pathogen resistance pathways (Figure 4), defense-associated plant hormone signaling (Figure 4), and WRKY TFs (Figure 5) were significantly enriched in *AtMYB12*-OX hairy roots. SA and JA play essential roles in plant defense against different pathogens (Yang et al., 2019; Chen et al., 2020). WRKY TFs are among the largest families of transcriptional regulators and contribute to various plant processes, including disease defense (Wani et al., 2021). Some WRKYs like AtWRKY33 could regulate SA/JA biosynthesis while others are regulated by SA/JA signaling (Birkenbihl et al., 2012; Wani et al., 2021). We speculate that AtMYB12 activated the SA/JA-WRKY network that contributes to the pathogen defense responses in *G. inflata* hairy roots.

Metabolic engineering offers an excellent approach for producing various bioactive, health-promoting phytochemicals in plants. This study underscores the importance of metabolic engineering for enhancing accumulation of valuable metabolites, such as licorice chalcones in *G. inflata*, through heterologous expression of a known flavonoid regulator. Metabolic pathways of non-model plants are relatively less studied. Transcriptomic and genomic resources will help unravel the biosynthetic pathways in non-model plants, such as *G. inflata*, and aid the bioengineering of bioactive compounds.

## Data availability statement

The data presented in the study are deposited in the NCBI repository, accession number: PRJNA842240.

## Author contributions

ZW and YLL performed the experiments, analyzed the data, and wrote the article. SS analyzed the RNA-seq data. RL, SP, YW, YQL, and LY evaluated the experiments and revised the article. YW, YQL, and LY initiated and supervised the project. All authors contributed to the article and approved the submitted version.
