# Supplementary material for: Metabolic engineering to enhance the accumulation of bioactive flavonoids licochalcone A and echinatin in Glycyrrhiza inflata (Licorice) hairy roots
Source: Front Plant Sci. 2022 Aug 18;13:932594. doi: 10.3389/fpls.2022.932594 (PMC9434314; doi:10.3389/fpls.2022.932594)

Supplementary Figure 1. Molecular analysis empty vector (EV) and *AtMYB12*-OX hairy root lines. *rolB*, protein-tyrosine phosphatase; *rolC* cytokinin-beta-glucosidase; *virC*, virulence protein; *NPTII*, neomycin phosphotransferase *II*.

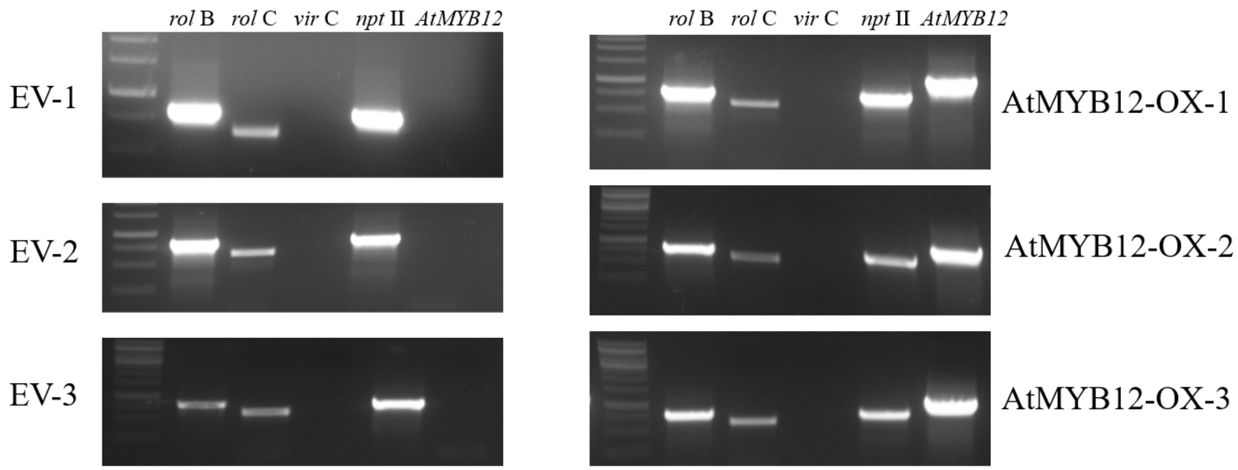

Supplementary Figure 2. The alignment matrices of RNAseq reads. The Y-axis represents the libraries while X-axis represents the number of reads in million.

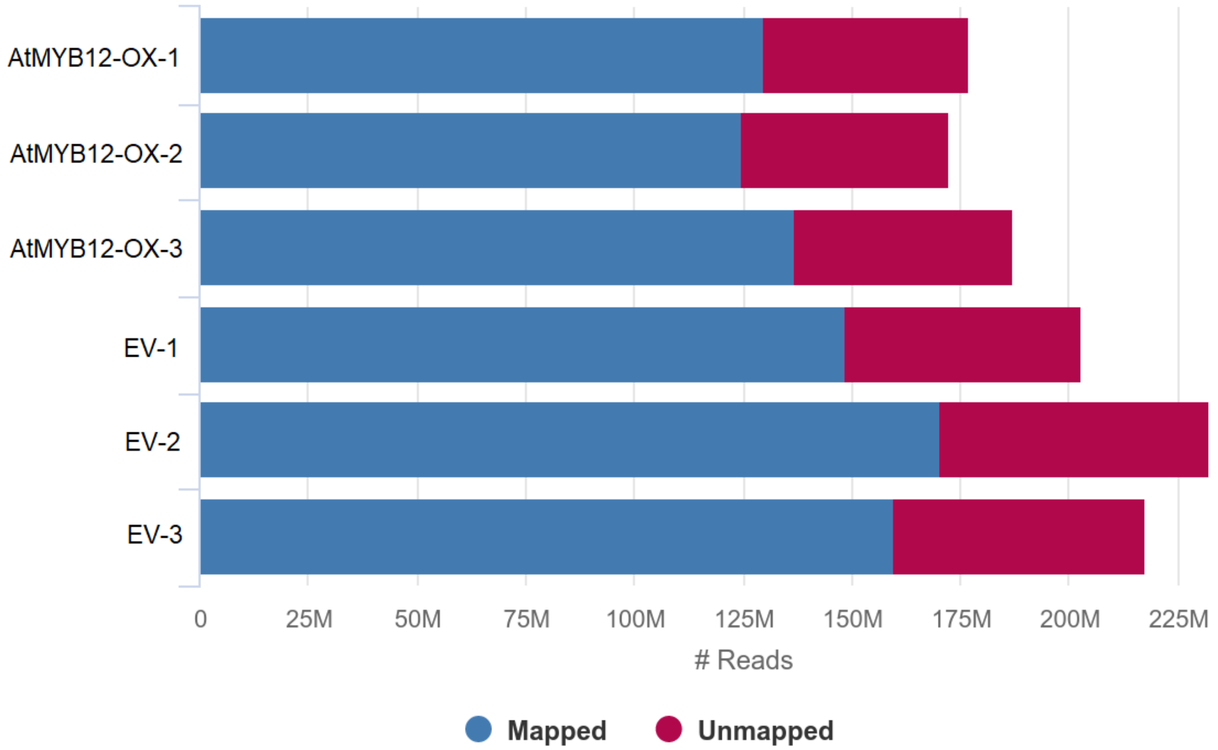

Supplementary Figure 3. Nucleotide sequence analysis of *G. inflata* and *G. uralensis* CHS1 promoters. Red arrow lines indicate primers used for promoter cloning. Red box indicates translational start codon (ATG).

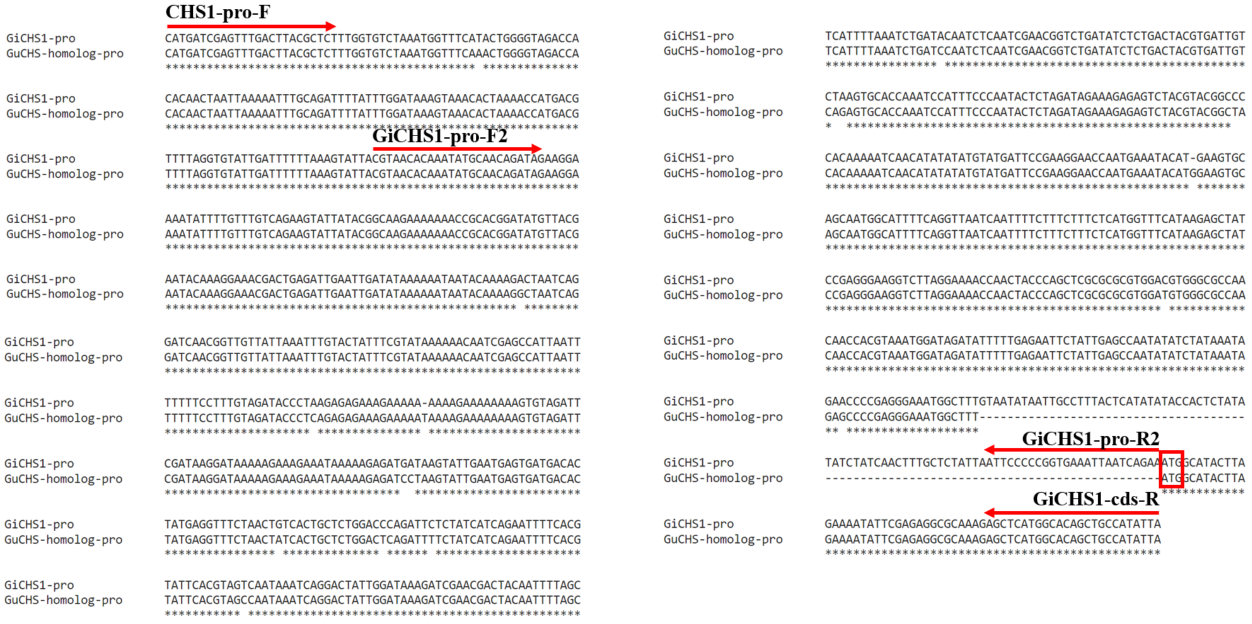

Supplement: Supplementary file 1 [file Data_Sheet_1.PDF]
